# Resting heart rate and impaired glucose regulation in middle-aged and elderly Chinese people: a cross-sectional analysis

**DOI:** 10.1186/s12872-017-0675-2

**Published:** 2017-09-13

**Authors:** Zhen Yang, Weiwei Zhang, Lingfei Zhu, Ning Lin, Yixin Niu, Xiaoyong Li, Shuai Lu, Hongmei Zhang, Xuanchun Wang, Jie Wen, Guang Ning, Li Qin, Qing Su

**Affiliations:** 1 Department of Endocrinology, Xinhua Hospital, Shanghai Jiaotong University School of Medicine, 1665 Kongjiang Road, Shanghai, 200092 China; 2 Department of Endocrinology, Xinhua Hospital Chongming Branch, Shanghai Jiaotong University School of Medicine, Shanghai, China; 3 Institute of Endocrinology and Diabetes, Department of Endocrinology and Metabolism, Huashan Hospital, Fudan University, Shanghai, China; 4 Shanghai Institute of Endocrinology and Metabolism, Department of Endocrine and Metabolic Diseases, Shanghai Clinical Center for Endocrine and Metabolic Diseases, Ruijin Hospital, Shanghai Jiao Tong University School of Medicine, Shanghai, China; 5 The Key Laboratory of Endocrine Tumors and the Division of Endocrine and Metabolic Diseases, E-Institute of Shanghai Universities, Shanghai, China

## Abstract

**Background:**

Elevated resting heart rate (RHR) has been reported to be associated with metabolic syndrome and type 2 diabetes. The aim of this study was to explore whether a positive relationship exists between RHR and impaired glucose regulation (IGR) among middle-aged and older Chinese individuals.

**Methods:**

We conducted a cross-sectional analysis that included a total of 9898 subjects (3194 men and 6704 women) in a Chinese population. The RHRs were derived from ECG recordings, and the subjects were stratified based on RHR quartiles.

**Results:**

RHR levels were significantly higher in the subjects with isolated impaired fasting glucose (i-IFG), isolated impaired glucose tolerance (i-IGT), IFG + IGT and diabetes than in those with normal glucose regulation. When multivariate logistic regression analyses were performed, the odds ratios were substantially higher for the subjects with IGR (odds ratio 2.19, 95% confidence interval 1.85–2.58) in the fourth RHR quartile compared with those in the first quartile after adjustment for potential confounding covariates, and the corresponding OR for the combined IGR and type 2 diabetes group was 2.56 (95% CI 2.20–2.98, *p* < 0.001). Multiple regression analyses demonstrated that RHR was significantly associated with fasting plasma glucose, 2-h OGTT plasma glucose and A1c.

**Conclusions:**

Our cross-sectional findings provide evidence that high RHR is associated with existing IGR among middle-aged and older Chinese individuals.

## Background

Elevated resting heart rate has been demonstrated to be associated with type 2 diabetes [1], metabolic syndrome [2, 3], cardiovascular disease, and all-cause mortality [4, 5]. As a global index of the influence of the autonomic nervous system on the heart, an elevated RHR can be considered as a reflex in response to autonomic imbalance, which refers to excessive sympathetic activity and too little parasympathetic activity [6]. Insulin resistance plays pivotal roles in the early development of hyperlipidemia, hyperglycemia, central adiposity, and atherosclerosis [7]. Furthermore, it is well documented that hyperinsulinism itself can trigger sympathetic nervous system activity [8, 9].

Although studies have clearly demonstrated the value of RHR in predicting the risk of mortality as well as the incidence of metabolic diseases, the influence of RHR on glucose metabolism has not been fully elucidated. Previous studies have confirmed that RHR is significantly associated with type 2 diabetes independently of conventional confounders [1, 10]. Most recently, Lawson et al. investigated the correlation of autonomic imbalance as measured by RHR with heart rate variability and metabolic risk outcomes and found that RHR and HRV, along with sex, age, and smoking were significant predictors of hyperglycemia and a diagnosis of diabetes within 12 years of follow-up [11].

However, to date, a very limited number of studies have reported conflicting data on the relationship between heart rate and glucose metabolism regulation [12–16]. In the present study, we sought to evaluate the association between the resting heart rate and states of glucose metabolism in a cross-sectional study of Chinese individuals aged 40–70 years.

## Methods

### Study population

The present study is a part of the risk evaluation of cancers in Chinese diabetic individuals, i.e., the longitudinal (REACTION) study that was a population-based cross-sectional study among middle-aged and elderly Chinese individuals aged 40–70 years in 25 communities across mainland China, conducted from 2011 to 2012. The details of the study design have been described previously [17, 18]. Briefly, all studied individuals came from the Chongming District in Shanghai, China. A total of 9930 eligible subjects were recruited. After excluding those who did not have resting heart rate measurements (*n* = 32), 9898 subjects (3194 men and 6704 women) were found to be eligible for the present analysis. The study protocol was approved by the Ethics Committee of Xinhua Hospital Affiliated to Shanghai Jiaotong University School of Medicine. Written informed consent was obtained from all of the participants.

### Data collection

The baseline data were collected by trained health workers via a standardized questionnaire during interviews. The measurements of weight, height, waist circumference, and blood pressure have been described previously. Smoking (current, former, or never) and alcohol drinking (current, former, or never) were assessed using an interview preceding the physical examination. The physical activity levels were classified as low, moderate, or high according to the International Physical Activity Questionnaire scoring protocol. BMI was calculated as weight in kilograms divided by the square of height in meters. RHR was measured and calculated from ECG recordings after subjects had ≥ 30 min rest and had been in the supine position ≥ 5 min.

All subjects were assessed after overnight fasting for at least 10 h. Overnight fasting and 2-h OGTT blood samples were collected. The fasting glucose, glucose 2 h after oral glucose tolerance test, triglycerides, total cholesterol (TC), high-density lipoprotein (HDL) cholesterol and low-density lipoprotein (LDL) cholesterol were measured with an automatic analyzer (Hitachi 7080; Tokyo, Japan). The homeostasis model assessment of insulin resistance (HOMA-IR) was calculated according to the equation described by Matthews et al. [19]. The abbreviated Modification of Diet in Renal Disease formula recalibrated for the Chinese was used to estimate the glomerular filtration rates, which are expressed in milliliters per minute per 1.73 m^2^ as follows: estimated glomerular filtration rate (eGFR) = 186 × [serum creatinine × 0.011]^-1.154^ × [age]^-0.203^ × [0.742 if female] × 1.233, where serum creatinine is expressed as micromoles per liter and 1.233 is the adjustment coefficient for the Chinese [20].

### Impaired glucose regulation and type 2 diabetes

Impaired glucose regulation was defined as IFG (i.e., a fasting plasma glucose level ≥ 6.1 and < 7.0 mmol/l) and/or IGT (2-h OGTT plasma glucose level ≥ 7.8 and < 11.1 mmol/l). Isolated IFG was defined as a fasting plasma glucose ≥ 6.1 mmol/l and < 7.0 mmol/l and a 2-h OGTT plasma glucose < 7.8 mmol/l. Isolated IGT was defined as a 2-h OGTT plasma glucose ≥ 7.8 mmol/l and < 11.1 mmol/l and a fasting glucose < 6.1 mmol/l. IFG/IGT was defined by a fasting plasma glucose between 6.1 mmol/l and 6.9 mmol/l and a 2-h OGTT plasma glucose of 7.8 mmol/l to 11.0 mmol/l. Type 2 diabetes was diagnosed according to the 1999 World Health Organization criteria (fasting plasma glucose level ≥ 7.0 mmol/l and/or a 2-h OGTT plasma glucose level ≥ 11.1 mmol/l or the current use of antidiabetic medication). A fasting glucose level lower than 6.1 mmol/l and a 2-h OGTT plasma glucose level below 7.8 mmol/l were defined as normal glucose regulation (NGR).

### Statistical analysis

The statistical analyses were performed with the SPSS Statistical Package (version 13.0; SPSS Inc., Chicago, IL). The participants’ characteristics are presented according to the RHR quartiles, which were defined as follows: < 71, 71–78, 79–86 and > 86 bpm. Analyses of covariance (general linear regression model) were applied for continuous variables, and multivariate logistic regression analyses were applied for the categorical variables for the comparisons according to the RHR quartiles. A multivariable stepwise regression analysis was used to investigate the association of RHR with cardiovascular and metabolic related parameters. A multivariate logistic regression model was used to evaluate the ORs and 95% CIs of having IGR or IGR + T2DM for each quartile of RHR compared with the lowest quartile with adjustments for potential confounding covariates. Finally, a multiple linear regression analysis was performed to determine the associations of fasting glucose, 2-h OGTT plasma glucose and A1c with the other related variables. When appropriate, natural log-transformed values were used for the analyses. *P* values < 0.05 were considered statistically significant.

## Results

The general clinical characteristics of the study population are presented in Table 1. Across the resting heart rate quartiles, the subjects with the higher resting heart rates were more likely to be of lower age, have smaller waist circumferences, have high lipid profiles and have chronic systemic inflammation. These subjects also tended to have an adverse glucose metabolism profile. Additionally, these subjects were less likely to be male, smokers and alcohol drinkers than those in the lower resting heart rate quartiles.

**Table 1:** Participants’ characteristics according to resting heart rate quartiles

Variable	RHR quartile (bpm)				*P* value
	Q1 < 71	Q2 71–78	Q3 79–86	Q4 > 86	
n	2166	2691	2563	2478	
Mean RHR, bpm	65 ± 4	75 ± 2	82 ± 2	96 ± 8	< 0.001
Males, n (%)	962 (44.4)	859 (31.9)	701 (27.4)	672 (27.1)	< 0.001
Age (years)	57.6 ± 7.5	56.1 ± 7.6	55.6 ± 8.0	55.2 ± 8.3	< 0.001
WC (cm)	85.3 ± 11.9	84.9 ± 10.2	84.7 ± 9.9	83.7 ± 10.4	< 0.001
Waist/hip ratio	0.9 ± 0.1	0.9 ± 0.1	0.9 ± 0.2	0.9 ± 0.1	0.002
BMI (kg/m^2^)	24.7 ± 3.3	24.7 ± 3.4	24.8 ± 7.8	24.3 ± 3.5	0.001
SBP (mm Hg)	129 ± 20	130 ± 18	132 ± 20	132 ± 19	< 0.001
DBP (mm Hg)	78 ± 10	79 ± 10	81 ± 10	83 ± 10	< 0.001
FPG (mmol/L)	6.1 ± 1.4	6.1 ± 1.6	6.5 ± 1.51	6.6 ± 2.2	< 0.001
2hPPG (mmol/L)	8.0 ± 3.5	8.3 ± 3.7	8.7 ± 3.7	9.7 ± 4.5	< 0.001
HbA1c	5.9 ± 0.9	6.0 ± 1.0	6.0 ± 1.0	6.1 ± 1.2	< 0.001
Fasting insulin (μU/ml)	6.0 (4.2–8.4)	6.5 (4.7–8.9)	6.8 (4.9–9.5)	7.0 (5.0–9.7)	< 0.001
HOMA-IR	1.6 (1.2–2.4)	1.7 (1.3–2.6)	1.8 (1.3–2.7)	2.0 (1.4–2.9)	< 0.001
TC (mmol/L)	4.6 ± 1.0	4.6 ± 1.0	4.6 ± 1.1	4.8 ± 1.1	< 0.001
TG (mmol/L)	1.3(0.9–1.9)	1.3 (0.9–1.9)	1.4 (1.0–2.1)	1.5 (1.0–2.2)	< 0.001
LDL-c (mmol/L)	2.6 ± 0.7	2.6 ± 0.8	2.6 ± 0.8	2.7 ± 0.8	0.022
HDL-c (mmol/L)	1.22 ± 0.31	1.22 ± 0.31	1.21 ± 0.31	1.25 ± 0.33	< 0.001
eGFR (ml min^−1^ 1.73 m^−2^)	124.5 ± 25.6	125.8425.4	126.5 ± 25.8	124.1 ± 25.3	0.001
CRP (mg/L)	1.3 (0.5–3.4)	1.4 (0.5–3.4)	1.5 (0.6–3.7)	1.6(0.7–3.8)	< 0.001
Current smokers, n (%)	379 (17.5)	351 (13.0)	290 (11.3)	270 (10.9)	< 0.001
Current drinkers, n (%)	247 (11.4)	286 (10.6)	242 (9.4)	226 (9.1)	0.033
IGR, n (%)	666 (30.8)	887 (33.0)	927 (36.2)	935 (37.7)	< 0.001
Type 2 diabetes, n (%)	484 (22.4)	578 (21.5)	646 (25.2)	817 (33.0)	< 0.001

The values are presented as the means ± the SDs, the medians (interquartile ranges) or the numbers (proportions)

*RHR* resting heart rate, *bpm* beats/min, *WC*, waist circumference, *BMI* body mass index, *SBP* systolic blood pressure, *DBP* diastolic blood pressure, *FPG* fasting plasma glucose, *2hPPG* 2-h post-load plasma glucose, *HbA1c* hemoglobin A1c, *TC* total cholesterol, *TG* triglycerides, *LDL* low-density lipoprotein-cholesterol, *HDL* high-density lipoprotein-cholesterol, *eGFR* estimated glomerular filtration rate, *CRP* C reactive protein, *IGR* impaired glucose regulation

Table 2 presents the results of regression analyses of resting heart rate with other cardiovascular and metabolic related parameters. In both the simple and multiple adjusted linear regression analyses, resting heart rate was positively associated with fasting plasma glucose, 2-h OGTT plasma glucose, A1c, HOMA-IR, systolic blood pressure, diastolic blood pressure, and triacylglycerol and negatively associated with BMI and waist circumference.

**Table 2:** Adjusted regression coefficients for resting heart rate according to cardiovascular- and metabolism-related parameters

	Model 1		Model 2		Model 3	
	ß (SEM)	*P* value	ß (SEM)	*P* value	ß (SEM)	*P* value
FPG	0.170 (0.143)	< 0.001	0.164 (0.144)	< 0.001	0.167 (0.145)	< 0.001
2hPPG	0.196 (0.051)	< 0.001	0.198 (0.051)	< 0.001	0.197 (0.051)	< 0.001
HbA1c	0.190 (0.218)	< 0.001	0.187 (0.220)	< 0.001	0.188 (0.220)	< 0.001
Fasting insulin	0.049 (0.019)	< 0.001	0.046 (0.021)	< 0.001	0.041 (0.021)	< 0.001
HOMA-IR	0.023 (0.049)	0.031	0.026 (0.050)	0.017	0.027 (0.050)	0.014
SBP	0.140 (0.009)	< 0.001	0.140 (0.009)	< 0.001	0.141 (0.009)	< 0.001
DBP	0.276 (0.016)	< 0.001	0.277 (0.016)	< 0.001	0.277 (0.016)	< 0.001
BMI	− 0.037 (0.027)	0.003	− 0.025 (0.029)	0.035	− 0.025 (0.029)	0.039
WC	− 0.086 (0.014)	< 0.001	− 0.054 (0.027)	0.014	− 0.057 (0.027)	0.010
TG	0.042 (0.094)	< 0.001	0.046 (0.099)	< 0.001	0.049 (0.100)	< 0.001
HDL-c	0.014 (0.414)	0.213	0.013 (0.415)	0.227)	0.017 (0.423)	0.116

Model 1: adjusted for age and sex; Model 2: further adjusted for smoking, drinking, and physical activity; Model 3: further adjusted for CRP and eGFR

Resting heart rate levels were significantly higher in the subjects with isolated IFG, isolated IGT, combined IFG and IGT, and type 2 diabetes compared with the subjects with normal glucose regulation (79.3, 80.2, 82.2, and 83.1 bpm, respectively, vs. 77.5 bpm, all *p* < 0.01) after adjusting for age and sex. The risk of having impaired glucose regulation increased progressively across the lowest to the highest quartiles of resting heart rate with ORs of 1.44 (95% CI 1.24–1.68), 1.93 (95% CI 1.66–2.25), and 2.19 (95% CI 1.85–2.58), respectively (P_*trend*_ < 0.001) after adjusting for sex, age, smoking, alcohol drinking, physical activity, BMI, waist circumference, SBP, DBP, and lipid profiles. For eGFR and CRP, the OR of have both impaired glucose regulation and type 2 diabetes was 2.56 (95% CI 2.20–2.98) after controlling for all the covariates in model 4 (Table 3).

**Table 3:** ORs for the risk of impaired glucose regulation and type 2 diabetes by resting heart rate quartile

	Resting heart rate quartiles (bpm)				P for trend
	Q1 < 72	Q2 72–79	Q3 80–86	Q4 > 86	
IGR					
Model 1	1	1.43 (1.25–1.64)	1.96 (1.72–2.24)	2.41 (2.09–2.78)	< 0.001
Model 2	1	1.48 (1.28–1.72)	2.03 (1.76–2.35)	2.38 (2.04–2.78)	< 0.001
Model 3	1	1.47 (1.26–1.71)	2.01 (1.73–2.34)	2.30 (1.95–2.71)	< 0.001
Model 4	1	1.44 (1.24–1.68)	1.93 (1.66–2.25)	2.19 (1.85–2.58)	< 0.001
IGR + T2DM					
Model 1	1	1.60 (1.41–1.80)	2.28 (2.02–2.57)	2.80 (2.46–3.18)	< 0.001
Model 2	1	1.64 (1.43–1.87)	2.35 (2.06–2.67)	2.79 (2.42–3.20)	< 0.001
Model 3	1	1.64 (1.43–1.89)	2.33 (2.03–2.68)	2.75 (2.37–3.19)	< 0.001
Model 4	1	1.60 (1.39–1.84)	2.20 (1.91–2.53)	2.56 (2.20–2.98)	< 0.001

Model 1: adjusted for sex and age; Model 2: further adjusted for smoking, alcohol drinking, and physical activity; Model 3: further adjusted for BMI, waist circumference, SBP, DBP, and lipid profiles; Model 4: further adjusted for eGFR and CRP

To further understand the influence of resting heart rate on the prevalence of impaired glucose regulation and type 2 diabetes, multiple regression analyses were performed. The analyses revealed that resting heart rate remained significantly associated with fasting plasma glucose, 2-h post-load plasma glucose and A1c (Table 4) after adjusting for all potentially confounding covariates.

**Table 4:** Multiple linear regression analysis assessing the associations of fasting plasma glucose, 2-h OGTT plasma glucose and A1c with RHR in men and women

Dependent variable	Variable	B	SE	Standardized B	*p* value
Men					
FPG	Age	0.026	0.003	0.112	< 0.001
	HOMA-IR	0.191	0.007	0.291	< 0.001
	RHR	0.022	0.002	0.136	< 0.001
(R^2^ = 0.169; F-value = 115.6), p < 0.001					
2-h PPG	Age	0.069	0.004	0.141	< 0.001
	HOMA-IR	0.356	0.018	0.238	< 0.001
	RHR	0.049	0.003	0.175	< 0.001
(R^2^ = 0.572; F-value = 113.8), p < 0.001					
HbA1c	Age	0.022	0.002	0.0161	< 0.001
	HOMA-IR	0.099	0.004	0.259	< 0.001
	RHR	0.007	0.002	0.071	< 0.001
(R^2^ = 0.121; F-value = 73.8), p < 0.001					
Women					
FPG	Age	0.030	0.002	0.109	< 0.001
	HOMA-IR	0.177	0.007	0.285	< 0.001
	RHR	0.017	0.003	0.144	< 0.001
(R^2^ = 0.160; F-value = 118.6), p < 0.001					
2-h PPG	Age	0.069	0.005	0.148	< 0.001
	HOMA-IR	0.361	0.017	0.237	< 0.001
	RHR	0.055	0.004	0.182	< 0.001
(R^2^ = 0.612; F-value = 114.8), p < 0.001					
HbA1c	Age	0.025	0.002	0.0173	< 0.001
	HOMA-IR	0.102	0.005	0.263	< 0.001
	RHR	0.007	0.003	0.081	< 0.001
(R^2^ = 0.152; F-value = 77.9), p < 0.001					

*FPG* fasting plasma glucose, *2hPPG* 2-h post-load plasma glucose, *RHR* resting heart rate

## Discussion

The novel finding of the present study is the independent association of a higher resting heart rate with impaired glucose regulation. Additionally, an elevated resting heart rate was also significantly associated with increased insulin resistance.

An elevated resting heart rate level has been found to be an independent predictor of cardiovascular and all-cause mortality [4, 21, 22]. Additionally, recent studies have indicated that a relatively high HR has direct detrimental effects on the progression of coronary atherosclerosis [23–26]. Clinical trial data have confirmed that HR reduction itself has a good effect on subjects with cardiovascular disease [4]. Furthermore, most recent studies have provided evidence that RHR is an independent risk factor of type 2 diabetes and metabolic syndrome [1, 3]. Whereas the above studies support the role of RHR in predicting the risks of mortality and the prevalence of metabolic diseases, in this study, we demonstrated that higher resting heart rate was a good indicator of an increased risk of impaired glucose regulation in middle-aged and elderly Chinese individuals, independent of potential confounders.

In line with previous studies [27–29], we also found that there was a significant association between RHR and fasting plasma insulin levels. Furthermore, multivariable stepwise regression analysis revealed that a higher RHR was an independent risk factor for insulin resistance. The results are consistent those of previous studies [27, 30] that have stated that elevated RHR levels are markedly correlated with increased insulin resistance. Resting heart rate is largely determined by the activity of the autonomic nervous system and hemodynamic states and has been recognized as an integrated index of the heart that is influenced by the autonomic nervous system. In a sense, the RHR is related to sympathetic activity or autonomic imbalance. Previous studies have suggested that there are links between sympathetic nervous activity, hyperinsulinemia and insulin resistance [31–33].

Impaired glucose regulation, also referred to as prediabetes, is associated with a relatively high risk for the future development of diabetes [34]. IGR is a highly heterogeneous metabolic state, both with respect to its pathogenesis and the prediction of disease [35]. IGR should not be viewed as a clinical entity in its own right but rather as a risk factor for diabetes and cardiovascular disease. Furthermore, it has been well documented that IGR is associated with hypertension, dyslipidemia with high triglycerides and/or low HDL cholesterol, and visceral obesity. Additionally, substantial epidemiological evidence indicates that the onsets of cardiovascular disease and type 2 diabetes can be delayed or prevented via lifestyle modifications. Therefore, it is critical to clarify the mechanisms underlying impaired glucose regulation and to seek effective management measures for all modifiable risk factors. In the present study, after controlling for potential cofounders, RHR emerged as an independent risk factor for IGR. Additionally, we also demonstrated that resting heart rate was significantly associated glucose metabolism indices (including fasting plasma glucose, 2-h post-load plasma glucose and A1c) in a multivariate analysis. Moreover, emerging data have established that HR reduction therapy has a beneficial influence on patients with cardiovascular disease. In view of these facts, it is plausible to consider whether RHR alone should become a novel target of clinical intervention for abnormal glucose metabolism just as it is for cardiovascular disease.

This cross-sectional study evaluated the relationship between RHR and impaired glucose regulation in a large sample of the Chinese population. Most potential confounders were carefully controlled, which limited the possibility of residual confounding effects. Furthermore, in our study, RHR data were collected via echocardiography, which enabled a precise RHR measurement. Our study does have several limitations. Given the cross-sectional nature of the study design, a cause-effect relationship between RHR and impaired glucose regulation could not be inferred. Further prospective study is needed to dissect the potential pathophysiological mechanism of this correlation. Additionally, the data were obtained from middle-aged and older people, and the gender proportions were unbalanced, which may limit the extrapolation of the results to a more general population. Moreover, this study used a single heart rate measurement, which was derived from ECG and might not represent the true resting heart rate. It would be more appropriate to have multiple resting heart rate measurements and to use the heart rate trend or at least an average heart rate measurement for the analysis.

## Conclusions

In summary, our findings suggested that elevated resting heart rate is strongly and independently associated with an increased risk of having impaired glucose regulation. As a familiar and accessible clinical variable, resting heart rate is a simple measurement that can be used to monitor abnormal glucose regulation in the population. Our results may have important public health implications. However, longitudinal studies are warranted to determine the clinical significance of these findings.

## References

[1] Carnethon MR, Yan L, Greenland P, Garside DB, Dyer AR, Metzger B, Daviglus ML (2008). Resting heart rate in middle age and diabetes development in older age. Diabetes Care.

[2] Rogowski O, Steinvil A, Berliner S, Cohen M, Saar N, Ben-Bassat OK, Shapira I (2009). Elevated resting heart rate is associated with the metabolic syndrome. Cardiovasc Diabetol.

[3] Jiang X, Liu X, Wu S, Zhang GQ, Peng M, Wu Y, Zheng X, Ruan C, Zhang W (2015). Metabolic syndrome is associated with and predicted by resting heart rate: across-sectional and longitudinal study. Heart.

[4] Jouven X, Empana JP, Schwartz PJ, Desnos M, Courbon D, Ducimetière P (2005). Heart-rate profile during exercise as a predictor of sudden death. N Engl J Med.

[5] Fox K, Borer JS, Camm AJ, Danchin N, Ferrari R, Lopez Sendon JL, Steg PG, Tardif JC, Tavazzi L, Tendera M, Heart rate working group (2007). Resting heart rate in cardiovascular disease. J Am Coll Cardiol.

[6] Perski A, Olsson G, Landou C, de Faire U, Theorell T, Hamsten A (1992). Minimum heart rate and coronary atherosclerosis: independent relations to global severity and rate of progression of angiographic lesions in men with myocardial infarction at a young age. Am Heart J.

[7] Reaven GM (1988). Banting lecture 1988. Role of insulin resistance in human disease. Diabetes.

[8] Anderson EA, Hoffman RP, Balon TW, Sinkey CA, Mark AL (1991). Hyperinsulinemia produces both sympathetic neural activation and vasodilation in normal humans. J Clin Invest.

[9] Vollenweider P, Randin D, Tappy L, Jéquier E, Nicod P, Scherrer U (1994). Impaired insulin-induced sympathetic neural activation and vasodilation in skeletal muscle in obese humans. J Clin Invest.

[10] Kim DI, Yang HI, Park JH, Lee MK, Kang DW, Chae JS, Lee JH, Jeon JY (2016). The association between resting heart rate and type 2 diabetes and hypertension in Korean adults. Heart.

[11] Wulsin LR, Horn PS, Perry JL, Massaro JM, D'Agostino RB (2015). Autonomic imbalance as a predictor of metabolic risks, cardiovascular disease, diabetes, and mortality. J Clin Endocrinol Metab.

[12] Nagaya T, Yoshida H, Takahashi H, Kawai M (2010). Resting heart rate and blood pressure, independent of each other, proportionally raise the risk for type-2 diabetes mellitus. Int J Epidemiol.

[13] Grantham NM, Magliano DJ, Tanamas SK, Söderberg S, Schlaich MP, Shaw JE (2013). Higher heart rate increases risk of diabetes among men: the Australian diabetes obesity and lifestyle (AusDiab) study. Diabet Med.

[14] Zhang SY, Wu JH, Zhou JW, Liang Z, Qiu QY, Xu T, Zhang MZ, Zhong CK, Jiang W, Zhang YH (2016). Overweight, resting heart rate, and prediabetes/diabetes: a population-based prospective cohort study among inner Mongolians in China. Sci Rep.

[15] Wang L, Cui L, Wang Y, Vaidya A, Chen S, Zhang C, Zhu Y, Li D, Hu FB, Wu S, Gao X (2015). Resting heart rate and the risk of developing impaired fasting glucose and diabetes: the Kailuan prospective study. Int J Epidemiol.

[16] Carnethon MR, Golden SH, Folsom AR, Haskell W, Liao D (2003). Prospective investigation of autonomic nervous system function and the development of type 2 diabetes: the atherosclerosis risk in communities study, 1987–1998. Circulation.

[17] Ning G, Reaction Study Group (2012). Risk evaluation of cAncers in Chinese diabetic individuals: a lONgitudinal (REACTION) study. J Diabetes.

[18] Qin L, Yang Z, Gu H, Lu S, Shi Q, Xing Y, Li X, Li R, Ning G, Su Q (2014). Association between serum uric acid levels and cardiovascular disease in middle-aged and elderly Chinese individuals. BMC Cardiovasc Disord.

[19] Matthews DR, Hosker JP, Rudenski AS, Naylor BA, Treacher DF, Turner RC (1985). Homeostasis model assessment: insulin resistance and beta-cell function from fasting plasma glucose and insulin concentrations in man. Diabetologia.

[20] Liu X, Qiu X, Shi C, Huang H, Huang J, Li M, Lou T (2014). Modified glomerular filtration rate-estimating equations developed in asiatic population for chinese patients with type 2 diabetes. Int J Endocrinol.

[21] Palatini P, Julius S (2004). Elevated heart rate: a major risk factor for cardiovascular disease. Clin Exp Hypertens.

[22] Palatini P, Benetos A, Julius S (2006). Impact of increased heart rate on clinical outcomes in hypertension: implications for antihypertensive drug therapy. Drugs.

[23] Beere PA, Glagov S, Zarins CK (1984). Retarding effect of lowered heart rate on coronary atherosclerosis. Science.

[24] Kaplan JR, Manuck SB, Clarkson TB (1987). The influence of heart rate on coronary artery atherosclerosis. J Cardiovasc Pharmacol.

[25] Beere PA, Glagov S, Zarins CK (1992). Experimental atherosclerosis at the carotid bifurcation of the cynomolgus monkey. Localization, compensatory enlargement, and the sparing effect of lowered heart rate. Arterioscler Thromb.

[26] Huikuri HV, Jokinen V, Syvänne M, Nieminen MS, Airaksinen KE, Ikäheimo MJ, Koistinen JM, Kauma H, Kesäniemi AY, Majahalme S, Niemelä KO, Frick MH (1999). Heart rate variability and progression of coronary atherosclerosis. Arterioscler Thromb Vasc Biol.

[27] Festa A, D'Agostino R, Hales CN, Mykkänen L, Haffner SM (2000). Heart rate in relation to insulin sensitivity and insulin secretion in nondiabetic subjects. Diabetes Care.

[28] Feskens EJ, Kromhout D (1994). Hyperinsulinemia, risk factors, and coronary heart disease. The Zutphen elderly study. Arterioscler Thromb.

[29] Palatini P, Casiglia E, Pauletto P, Staessen J, Kaciroti N, Julius S (1997). Relationship of tachycardia with high blood pressure and metabolicabnormalities: a study with mixture analysis in three populations. Hypertension.

[30] Adachi H, Hashimoto R, Tsuruta M, Jacobs DR, Crow RS, Imaizumi T (1997). Hyperinsulinemia and the development of ST-T electrocardiographic abnormalities. An 11-year follow-up study. Diabetes Care.

[31] Scherrer U, Randin D, Tappy L, Vollenweider P, Jéquier E, Nicod P (1994). Body fat and sympathetic nerve activity in healthy subjects. Circulation.

[32] Hausberg M, Hoffman RP, Somers VK, Sinkey CA, Mark AL, Anderson EA (1997). Contrasting autonomic and hemodynamic effects of insulin in healthy elderly versus youngsubjects. Hypertension.

[33] Svensson MK, Lindmark S, Wiklund U, Rask P, Karlsson M, Myrin J, Kullberg J, Johansson L, Eriksson JW (2016). Alterations in heart rate variability during everyday life are linked to insulin resistance. A role of dominating sympathetic over parasympathetic nerveactivity?. Cardiovasc Diabetol.

[34] Alberti KG, Zimmet PZ (1998). Definition, diagnosis and classification of diabetes mellitus and its complications. Part 1:diagnosis and classification of diabetes mellitus provisional report of a WHO consultation. Diabet Med.

[35] Stefan N, Fritsche A, Schick F, Häring HU (2016). Phenotypes of prediabetes and stratification of cardiometabolic risk. Lancet Diabetes Endocrinol.

